# Isolated screw fixation of posterior wall fractures

**DOI:** 10.1007/s00402-026-06408-y

**Published:** 2026-07-03

**Authors:** Jan-Dierk Clausen, Hür Özbek, Tarek Omar-Pacha, Stephan Sehmisch, Axel Gänsslen

**Affiliations:** Department of Trauma Surgery, Hannover Medical School, Carl Neuberg Str. 1, Hannover, Germany

**Keywords:** Acetabular fracture, Posterior wall fracture, ORIF, Screw fixation, Long-term results

## Abstract

**Introduction:**

Posterior wall (PW) fractures are common acetabular fractures and are most often treated by open reduction and internal fixation. The gold-standard of surgical stabilization is screw fixation of large wall fragments with additional buttress plating. Some reports focussed on screw fixation alone presenting adequate results.

**Material and methods:**

From a total of 208 PW-fracture treated between 1972 and 2008, 57 patients were identified with open reduction and internal fixation (ORIF) using isolated screw fixation. These patients were analyzed regarding demographical data (patient age, sex), type of accident, injury mechanism, Injury Severity Score (ISS), concomitant pelvic ring injuries, associated injuries, perioperative parameters and long-term results.

**Results:**

44 patients were male and 13 were female with a mean age of 36.8 years. More than 90% sustained high-energy trauma with 34 sustaining multiple injuries or polytrauma. The mean Injury Severity Score was 11.5 points. All patients had fracture dislocations, which were reduced within 24 h after admission except one patient with secondary transfer. 11 patients presented with an initial sciatic nerve deficit. 29 patients had a single PW-fragment, 16 presented with two PW-fragments and in 12 patients marginal impactions were present. Surgery was performed in average after 7 days using the Kocher-Langenbeck approach. 24.6% femoral head lesions and 19.3% acetabular lesions were detected. All hips could be reconstructed anatomically. One patient died after severe head injury. 41 patients had follow-up of at least 12 months: 80.5% reported none or slight pain and 90.2% had good to excellent clinical results. 78% had no post-traumatic hip joint changes.

**Conclusion:**

Screw fixation alone can be considered as a rare but adequate treatment modality in selected posterior wall fractures, especially minor comminuted fractures with predominantly a large single posterior wall fragment.

## Introduction

Posterior wall fractures (PW) are of interest since more than 100 years. Initial reports focused on hip dislocations associated with posterior acetabular fractures.

These fractures were identified to be associated with relevant local concomitant inuries. Already, Waller described additional knee injuries, sciatic nerve lesions and femoral head (FH) fractures [47].

Analysis of consecutive data reveal a rate of 17% PW-fractures in the group of all acetabular fractures [1, 12, 13, 30, 37, 42, 47, 48]. PW-fractures were less common in elderly patients but present with a higher frequency, if surgery was performed [1, 18, 20, 27].

Letournel and Judet described the fracture morphology of PW-fractures more in detail and distinguished classical pure posterior PW-fractures and posterior-superior and posterior-inferior fractures [30]. The classical PW-fracture consists of a single pure posterior fragment involving the postero-lateral quadrangular area without involvement of the acetabular roof.

This classical fracture morphology presents as a near quadrangular area not extending to the whole posterior surface of the posterior column [7].

PW-fractures are commonly associated with relevant joint instability, either due to the fracture itself and/or due to accompanying avulsion injuries of the posterior capsule-labral complex. The combination of bony PW-defects with capsular disruption increases the risk of hip joint instability [30, 46]. PW involvement of < 20% are usually associated with a stable hip, while defect > 30% are usually treated surgically [6, 26, 34]. As PW-defects lead to increased superior peak loads, resulting in an increased the risk of secondary arthritis [38, 39, 43], surgical indications include unstable and incongruent joints, intraarticular fragments, progressive sciatic nerve lesions, associated femoral head fractures, acetabular marginal impactions and osteocartilaginous avulsion injuries and/or incarcerated fragments.

The typical treatment of PW-fractures consists of screw fixation of the wall fragment(s) followed by posterior column/wall neutralization plate stabilization, bridging the fragment zone [30].

Despite this recommendation, some biomechanical data [19, 31, 50] showed, that isolated screw fixation is formally adequate, while in comminuted fractures, an additional neutralization plate should be added [8, 11, 40, 49]. Additionally, some few clinical data found adequate results, when isolated screw fixation was used for PW-fractures [23].

During the last years several reports focused on isolated arthroscopic PW-fracture fixation and reported on case series with nine [51], six [3] and 13 patients [22] and presented adequate results.

## Material and methods

Between 01.01.1972 and 31.12.2008 a total of 1034 adult patients (age at least 14 years) were treated with acetabular fractures at our institution. 208 of these had a posterior wall fracture (incidence: 20.1%).

**Fig. 1:**
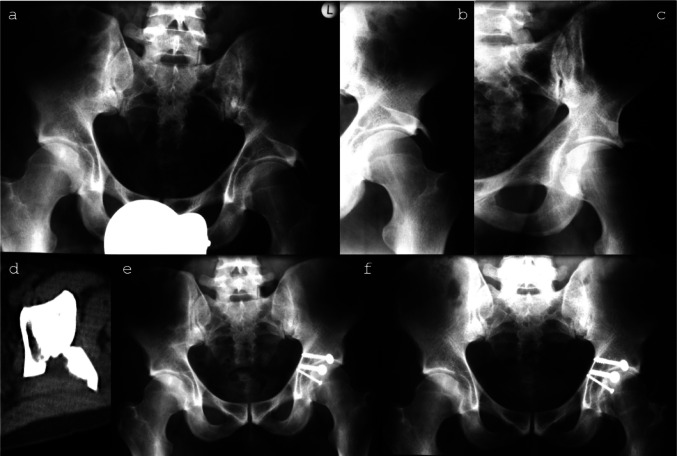
33 year-old male after MVA sustaining a moderate traumatic brain injury and a dashboard injury with a large posterior wall fracture without hip dislocation. The conventional x-rays (**a, b, c**) show the isolated wall fragment at its typical location posterior and superior to the acetabulum. CT confirmed. the single large fragment (**d**). After 10 days, ORIF was performed using the Kocher-Langenbeck approach and three lag screws were used (**e**). 3-year follow-up showed radiological uneventful healing. The clinical result was graded very good (Merle d´Aubigné Score 17 pt, slight groin pain, normal hip range of motion, no nerve deficit)

In 57 patients, open reduction and internal fixation (ORIF) was performed using isolated screw fixation. These 57 patients were analyzed regarding demographical data (patient age, sex), type of accident, injury mechanism, Injury Severity Score (ISS), concomitant pelvic ring injuries and associated injuries (see Fig. 1).

Traumatic brain injury (TBI) was graded according to the Glasgow Coma Scale (GCS) [44]. Accompanying chest and abdominal injuries were classified according to the Organ Injury Scaling (OIS) [36]. Additional pelvic ring injuries were analyzed alone and according to the complex pelvic trauma definition [4]. A fracture-related nerve damage was divided into primary and secondary (iatrogenic) nerve damage. Additional type of hip dislocation, time of reduction (hours), time of surgical stabilization, surgical approach, and surgery time with blood loss as well as damage to the femoral head (contusion, impaction, femoral head (FH) fracture), the acetabulum (contusion, impaction, comminution zones) and the presence of intra-articular fragments were recorded. The postoperative radiological result was graded according to Matta´s criteria [32].

The follow-up was recorded using a standardized documentation form [45] including pain analysis and neurological impairments [10]. The long-term functional outcome was classified according to the Merle d ‘Aubigné-score [33]. Assessment of the radiological result included follow-up x-ray examination for posttraumatic osteoarthritis changes according to (in: [45]), the presence of femoral head necrosis according to Ficat and Arlet [14] and the presence of heterotopic ossification according to Brooker [5]. In addition, secondary total hip replacement (THR) was documented. A radiological joint failure was defined as the presence of post-traumatic osteoarthritis grade 4 and/or the presence of post-traumatic femoral head necrosis grade 3 or 4 and/or the presence of heterotopic ossification of grade 4 and/or secondary THR.

Ethic approval was achieved from the Ethic Committee of Hannover Medical School: 12361-BO-K-2026. No funding was received for the study.

## Results

**Fig. 2:**
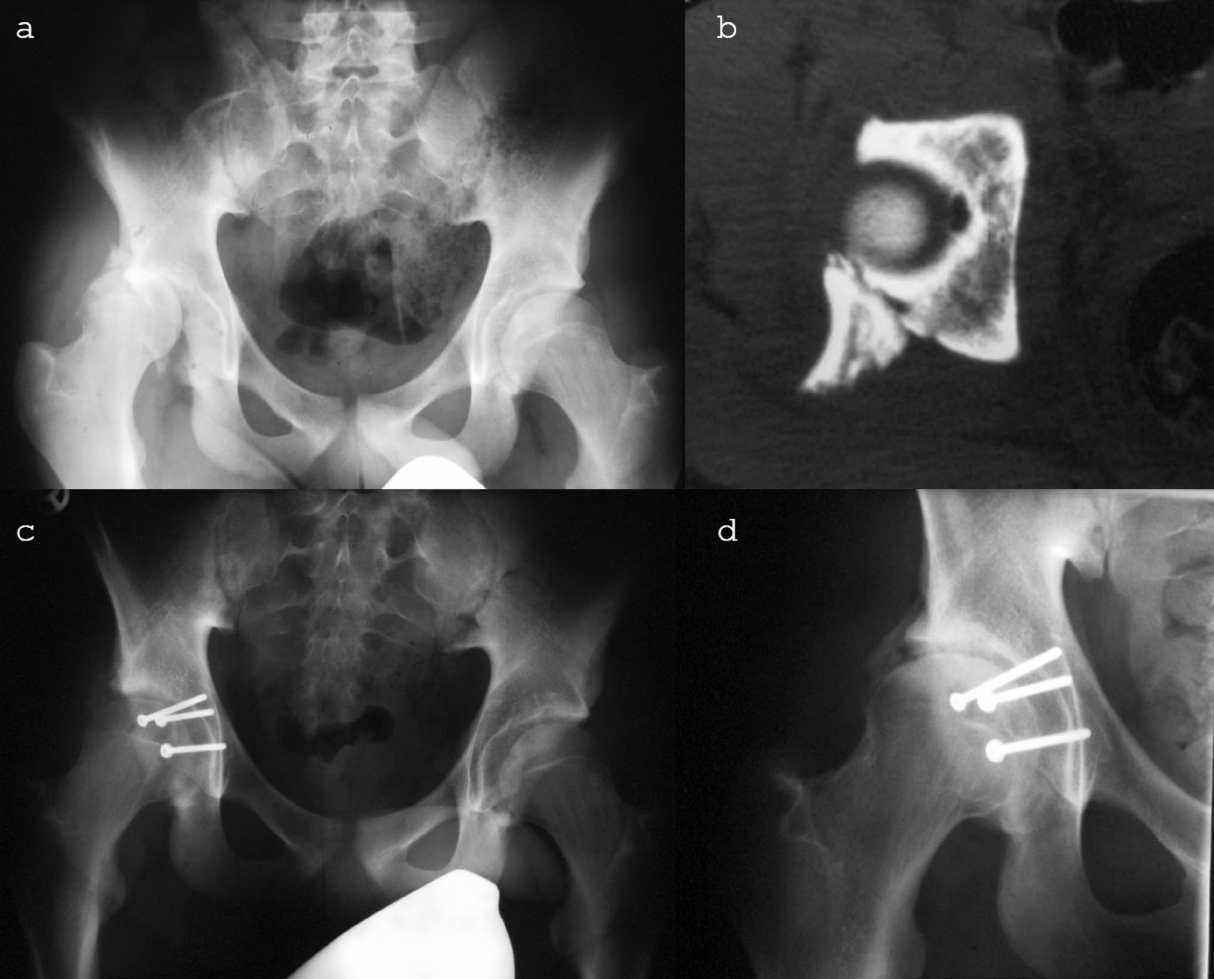
17 year-old male patient with a I° open tibia fracture and a posterior superior acetabular fracture dislocation (**a**) with two larger posterior wall fragments (**b**). ORIF was performed on day 13 with the Kocher-Langenbeck approach and screw stabilization asperformed with two screws in the proximal fragment and one screw for fixation of the more distal fragment (**c**). Intraoperatively a femoral head contusionwith slight marginal impaction was noted. 2-year follow-up showed radiological uneventful healing. The clinical result was graded excellent (Merle d´Aubigné Score 18 pt, no pain, normal hip range of motion, no nerve deficit)

57 patients with PW-fractures were identified with isolated PW screw fixation. This group of patients were treated between 1972 and 2002 (see Fig. 2).

### Patient demographics

44 patients were male and 13 were female. The mean age was 36.8 years (range 14–72 years).

39 patients were injured in a motor vehicle accident, three as truck drivers, eight in a motorcycle accident, one in a bicycle accident, five during a simple fall and two with other mechanisms. A high energy trauma was responsible in > 90% of patients.

In 22 patients, the acetabular PW-fractures was an isolated injury. 34 patients sustained multiple injuries and two were polytraumatized.

16 patients sustained a traumatic brain injury (TBI): 12 × I°, 3xII° and one III°. Nine patients presented with a chest trauma: eight with minor injuries, one moderate chest trauma. Concomitant abdominal or urogenital injuries were not observed. Five patients had upper extremity fractures and 20 patients had lower extremity fractures. An associated sciatic nerve injury was seen in 11 patients (19.3%).

The mean Injury Severity Score was 11.5 points (range: 9–34 points).

Two patients had an additional pelvic ring injury: one ipsilateral upper and lower anterior ring fracture, one contralateral type SI-joint injury with an associated upper and lower anterior ring fracture.

### Associated lower extremity injuries

A dashboard injury mechanism was suspected in 21 cases. The other patients had different specific injury mechanisms.

All patients had fracture dislocations: seven had a posterior, 49 a posterior-superior and one a central hip dislocation.

50 hips were reduced within eight hours. Five hips were reduced between 12 and 17 h. One patient, secondarily transferred, had reduction after 46 h. The mean reduction time was hours (range:0–46 h) and the median reduction time was within 1 h after admission.

One patients sustained a femoral shaft fracture, one a femoral neck fracture, three a lower leg fracture and six had ankle/foot fractures.

A distal patella pole avulsion fractures was seen in three, a tibial head fracture in three and an open knee injury in one patient.

Associated knee ligament injuries were observed in eight patients: 2 × postero-lateral knee instability, two ACL injuries, three PCL injuries and two MCL injuries.

### Sciatic nerve lesions

11 patients presented with an initial sciatic nerve deficit. Nine patients had a full nerve deficit, while two had a partial nerve deficit (peroneal part of the sciatic nerve).

One of these patients died and from the remaining 10 patients, six had follow up with five full recoveries and one partial recovery.

### Perioperative data and fracture characteristics

PW fractures were classified according to the 1996 AO/OTA compendium (J Orthop Trauma 1996:10 Suppl 1:v-ix, 1–154. Fracture and dislocation compendium. Orthopaedic Trauma Association Committee for Coding and Classification).

29 patients had a single PW fragment without an impaction. 16 presented with two PW fragments. In 12 patients beside one or two main fragments, marginal impactions were present. Three patients had undisplaced fractures and all other fracture were severely displaced (> 5 mm).

Surgery was performed in average after 7 days (range: 1–26 days). All patients were treated using the Kocher-Langenbeck approach.

The mean surgical time was 104 min (range: 40–270 min) and the mean expected blood loss was 323 ml (range: 50-1500 ml).

Femoral head lesions were detected intraoperatively in 14 patients (24.6%): 5 isolated FH contusions, one isolated FH impaction, 4 isolated FH fractures (Pipkin fracture) and four combined FH contusions and impactions with an additional Pipkin fracture.

Associated acetabular cartilage injuries were seen in 11 patients (19.3%): one cartilage contusion, nine marginal impactions and two combined acetabular cartilage contusions and impactions. In five cases a comminution zone was present. In 11 patients intraarticular fragments were observed.

All 57 patients had anatomic reduction based on standard x-rays and intraoperative findings. Onlyx marginal CT data were.

### Initial clinical course

Two patients developed superficial wound problems. None of these needed revision surgery. In one patient a re-dislocation occurred due to an overlooked intraarticular fragment. One patient developed wound hematoma, which resolved under conservative treatment. One patient died due to associated severe TBI (overall mortality rate: 1.7%).

The majority of patients performed partial weight bearing with 15 kg body weight for 6–12 weeks.

### Long-term results

Of 56 survivors, 41 patients had follow-up of at least 12 months (mean follow-up time: 56.8 months, range 13–239 months) (Fig. 1 and 2).

18 patients reported no pain and 15 patients slight pain. Moderate pain (six patients) and severe pain (two patients) were associated with joint degeneration and heterotopic bone formation in seven patients. Thus, 80.5% reported none or only slight pain at follow-up. The average subjective Visual Analog Scale pain score at follow-up was 17.73 points (0–92 points).

The mean Merle d ‘Aubigné score was 16.7 points (range: 10–18 points). According to the grading of the Merle d ‘Aubigné score, 43.9% of patients had a perfect functional result, 46.3% a good, and each 4.9% a moderate or poor functional outcome. Overall, the functional result was graded excellent and good in 90.2%. Of the four patients with moderate and poor functional results, all had moderate degenerative changes or relevant heterotopic ossification (grade 3 and 4).

A persistent nerve deficit was observed in one patient, while five had full recovery of their initial sciatic nerve lesion.

At follow-up 32 patients (78%) had no post-traumatic osteoarthritic changes of their hip joint. Five patients had mild changes (12.2%), three patients (7.3%) had moderate arthritis and one patient had severe joint destruction.

One patient developed severe FHN and had secondary THR 18 years after initial surgery.

Two patients developed heterotopic ossification grade II and IV, both with moderate and severe subjective pain.

A joint failure was diagnosed in 17.1% of the patients after an average of 83 months (range: 29–239 months).

## Discussion

Historically, screw fixation was the primary surgical method for unstable posterior wall fractures.

Griswold first described a surgical stabilization of a comminuted posterior rim fracture in a patient with an additional sciatic nerve palsy in 1929. A Sherman screw was used for fixation of a large posterior wall fragment [21]. Funsten et al. reported on one of 20 patients with an ivory pin fixation [16]. King et al. in 1941 often observed inadequate reduction (subluxation) of the femoral head, the lip fragment, or both, after conservative treatment and discussed, that surgical treatment may optimize the results with perfect anatomical reduction. An approach was described, comparable to the Kocher-Langenbeck incision, with wide exposure of the posterior wall area allowing fixation of this fragment using Matthews wires, nails, beef-bone screws, or vitallium screws, respectively [28].

It was the merit of Robert Judet and Emile Letournel to standardize the operative fixation technique of posterior wall fractures [25, 29, 30].

The combination of screw osteosynthesis of large PW-fragments with a posterior neutralization plate became gold-standard in treating PW-fractures [30].

Using this techniques adequate clinical and long-term radiological results can be achieved.

In a recent literature analysis, a high rate of anatomic reductions (85.7%) could be achieved. 80% of patients presented with excellent and good clinical results after one year, decreasing to 70% after 5 years. In contrast, the radiological results were constant, with slightly above 80% excellent and good results. The overall rate of osteoarthritic changes was 15.7% and the rate of secondary femoral head necrosis (FHN) was 9.3%. Rarely (2.6%) grade III-IV heterotopic ossification was reported [17].

Single screw fixation of isolated large posterior wall fragments, isolated fixation using spring-plates or isolated buttress plating is biomechanically adequate [15, 50], but additional application of a neutralization/buttress plate is generally recommended [30].

Some few reports are available for isolated screw fixation of posterior wall fractures.

In 2004, Im et al. reported on 15 patients with PW-fractures treated with cannulated screws alone [23]. All five patients with a single large fragment had excellent clinical results using the Merle d`Aubigné score. Of the 10 patients with comminuted PW-fractures, nine had excellent to good clinical results and one patient had a poor result after development of AVN of the femoral head. The follow-up time was at least after 2.3 years (range 2.3–4.2 years).

In a further analysis of 15 patients with a large with a single fragment and only moderate comminution of the posterior wall fracture area, 10 patients had excellent and very good clinical results (Merle d`Aubigné score 17 – 18 points) and four presented with a good result (15–16 points) after > 2 years [24].

In 2008, Berton Moed noted, that “Posterior wall fractures consisting of one large fragment may be stabilized satisfactorily with use of screws alone. However, the more conservative course of action is always to supplement screw fixation with a buttress plate. Fixation with use of screws alone is not indicated in comminuted fractures.” [35].

Qi et al. performed two fixation concepts. In less comminuted fractures with a large main posterior-superior fragment multiple screw fixation was performed, while in severely comminuted fractures with an posterior-inferior main fragment, reconstruction plating was advised [41]. In their series of 31 patients with PW-fractures, seven had isolated screw stabilization. No fixation failures were observed. Overall, a rate of 92% good to excellent results were observed in this specific treatment group.

In 2014, de Palma et al. reported on eight patients with a large single PW-fracture fragment stabilized with 2 (7 patients) or 3 (1 patient) lag screws without distinguishing the long-term clinical results of these patients from 34 patients with combined screw and plate fixation [9]. Of 11 patients with a single large PW-fracture, all had good to excellent clinical results after 2–10 years (Merle d´aubigné score).

Based on these data, screw fixation alone can be considered as a rare but adequate treatment modality in selected posterior wall fractures, especially minor comminuted fractures with predominantly a large single posterior wall fragment.

During recent years, some special reports focused on arthroscopic-assisted reduction and fixation of PW-fractures.

Aprato et al. reported on eight patients with arthroscopic-assisted screw fixation of posterior rim avulsion injuries, defined as a displaced single fragment, with less than 25% posterior wall involvement [3]. Using a standardized technique [2, 3], in all eight patients anatomic reduction could be achieved using two headless screws and the clinical results after at least one year was highly excellent with a mean modified Harris Hip Score of 98 points. No joint degeneration (heterotopic ossification (HO), AVN or posttraumatic osteoarthritis (ptOA)) nor fixation failure was observed in this group of young patients (mean age: 24 years).

Hwang et al. analyzed 13 patients (mean age 39 years, nine with fracture dislocation) with arthroscopic reduction and cannulated screw fixation (). Five patients had an additional femoral head (Pipkin-type) fracture. 11 of 13 patients had anatomic reduction (84.6%) and 12 of 13 patients had good to excellent clinical results after a mean of 21 months. No radiographic impairments were observed, e.g. HO, AVN or ptOA, fixation failure [22].

Overall, these data also indicate adequate fixation using screws in selected PW-fractures, even after arthroscopic surgery.

Our data show comparable results with a high rate of anatomical reductions and no fixation failures.

Long-term results were adequate with 80.5% reporting no or only slight pain at follow-up, a mean Merle d ‘Aubigné score of 16.7 points representing 90.2% excellent, very good and good clinical results.

In contrast to the mentioned arthroscopic analyses, we observed 22% joint problems, e.g. HO, AVN or ptOA with an overall joint failure rate of 17.1% after an average of 83 months.

A possible explanation is the longer follow-up period and a different indication and PW-fracture morphology. In our series, a nearly 25% rate of FH lesions including 5 Pipkin-type fractures and a 19.3% rate of relevant associated articular acetabular injuries were observed including relevant marginal impactions and fragment comminution.

Interestingly, our patient group was treated between 1972 and 2002, when surgical stabilization of acetabular fractures started without standards used today.

Despite these specific special features, the results of single screw fixation were promising.

## Conclusion

Posterior wall fractures are common acetabular fracture types and are the result of high energy trauma in the majority of patients. Therefore, significant injury to the acetabular cartilage and additional local injuries are often observed.

Open reduction and internal fixation with exclusively screws resulted in adequate rates of anatomical joint reconstruction and even adequate long-term results.

Screw fixation alone is considered an option in selected cases, especially with a single large PW-fragment.

## Data Availability

No datasets were generated or analysed during the current study.
